# Predictability of human mobility during the COVID-19 pandemic in the United States

**DOI:** 10.1093/pnasnexus/pgae308

**Published:** 2024-07-26

**Authors:** Michal Hajlasz, Sen Pei

**Affiliations:** Department of Computer Science, Columbia University, 500 W 120th St, New York, NY 10027, USA; Department of Environmental Health Sciences, Mailman School of Public Health, Columbia University, 722 W 168th St, New York, NY 10032, USA

**Keywords:** human mobility, predictability, human behavior, COVID-19, epidemics

## Abstract

Human mobility is fundamental to a range of applications including epidemic control, urban planning, and traffic engineering. While laws governing individual movement trajectories and population flows across locations have been extensively studied, the predictability of population-level mobility during the COVID-19 pandemic driven by specific activities such as work, shopping, and recreation remains elusive. Here we analyze mobility data for six place categories at the US county level from 2020 February 15 to 2021 November 23 and measure how the predictability of these mobility metrics changed during the COVID-19 pandemic. We quantify the time-varying predictability in each place category using an information-theoretic metric, permutation entropy. We find disparate predictability patterns across place categories over the course of the pandemic, suggesting differential behavioral changes in human activities perturbed by disease outbreaks. Notably, predictability change in foot traffic to residential locations is mostly in the opposite direction to other mobility categories. Specifically, visits to residences had the highest predictability during stay-at-home orders in March 2020, while visits to other location types had low predictability during this period. This pattern flipped after the lifting of restrictions during summer 2020. We identify four key factors, including weather conditions, population size, COVID-19 case growth, and government policies, and estimate their nonlinear effects on mobility predictability. Our findings provide insights on how people change their behaviors during public health emergencies and may inform improved interventions in future epidemics.

**Significance Statement**

Human mobility can shape the course of epidemics and may change in response to disease outbreaks. This behavior-epidemic feedback needs to be incorporated into next-generation predictive models for infectious diseases. However, a fundamental question remains: whether such mobility change can be predicted during disease outbreaks? Here we analyze human mobility driven by routine activities at the US county level and measure the predictability using an information-theoretic metric. We find that visits to residences under stay-at-home orders in spring 2020 were more predictable, while visits to other places had a higher predictability during summer 2020. Statistical analysis indicates that weather conditions, population size, COVID-19 case growth, and government policies had large influence on mobility predictability for all six place categories.

## Introduction

Human mobility is the fabric weaving together our society. Societal operations such as social, economic, and cultural exchanges depend critically on how people move around (1–4). Understanding patterns of human mobility is crucial for a variety of applications spanning epidemic control (5–14), social segregation (15–17), and disaster response (18–21). A plethora of studies have been devoted to discovering laws governing the mobility of individuals and population (1–4, 22–29). For instance, Brockmann et al. (4) discovered scaling laws of human mobility, showing that the distribution of traveling distances decays as a power law. Alessandretti et al. (1) found meaningful scales in day-to-day human mobility and showed that aggregating mobility at different scales gives rise to the power-law distribution of traveling distances. Schläpfer et al. (2) found that the number of visitors to any location decreases as the inverse square of the product of their visiting frequency and travel distance. These studies motivated a large body of research on predicting human mobility at various scales using novel computational methods (30–32).

In the context of infectious diseases, human mobility drives the growth and expansion of pathogens. Prior to the COVID-19 pandemic, there was a rich history of researchers using mobility data to study infectious disease dynamics, spanning diverse pathogens with different transmission modes [e.g. respiratory droplet/airborne (5–7) to vector-borne (8–10)] and across different geographic resolutions (within-city, country, and global). However, the use of mobility data in epidemic modeling essentially exploded during the COVID-19 pandemic (33–37), due to previously proprietary mobility data becoming publicly available for use in public health response. For example, Perofsky et al. (38) studied the impacts of human mobility on the citywide transmission dynamics of 18 respiratory viruses in pre- and post-COVID-19 pandemic years using mobile device location data; Pullano et al. (39) characterized the spatial connectivity of US counties and explored its implications for geographical disease dynamics using mobile phone-derived mobility data; Zhang et al. (40) employed aggregated foot traffic data and developed a behavior-driven epidemic model to predict neighborhood-level COVID-19 spread in New York City. Many epidemic models incorporated human mobility data to predict the progression of epidemics (6, 41–44). However, people may change their behaviors in response to disease outbreaks. It is a consensus that next-generation predictive models for infectious diseases need to incorporate the feedback between epidemic spread and behavioral changes (45, 46). Fundamentally, the success of this approach depends on whether we can accurately predict human mobility during disease outbreaks.

Many previous studies have explored the predictability of human mobility (47–51). Song et al. (47) showed that individual-level mobility trajectories are highly predictable. Lu et al. (50) used mobile phone call records to show that the theoretical maximum predictability of human mobility can be approached using Markov Chain based models. Chen et al. (48) found that colocation information from both social and nonsocial sources (i.e. colocation with acquaintances or strangers) contains predictive information of an individual's mobility pattern. Liu et al. (52) found that there is a switching phenomenon of spatiotemporal interaction patterns in cities, driven by different human mobility during the active state in the daytime and the sleeping state in the nighttime. Residents in larger cities tend to have a shorter sleeping time, which may lower the predictability of human mobility.

Despite these studies, whether the predictability of human mobility persisted during the COVID-19 pandemic remains unclear. Mobility patterns in foot traffic differ across different location types for day-to-day activities such as work, shopping, dining, and recreation, which in turn affect human contact rates and the clustering of contacts. Depending on the relative infection risk in those location types, people may change their mobility in specific place categories, either voluntarily or following government policies implementing nonpharmaceutical interventions (NPIs), such as stay-at-home orders, gathering restrictions, and business closures. For epidemic forecasting informed by mobility data, a fundamental question is to what degree these reactive mobility changes can be predicted. Human mobility data collected during the COVID-19 pandemic offer an opportunity to answer this question.

In this study, we analyzed mobility data measuring foot traffic (i.e. visits) to six place categories (i.e. residences, workplaces, transit stations, parks, groceries and pharmacies, and retail and recreation) in US counties from 2020 February 15 to 2021 November 23. We measured the predictability of mobility changes relative to the baseline prior to the pandemic in the six categories using an information-theoretic metric, permutation entropy (PE). PE is a robust measure for studying the complexity of a time series, introduced by Bandt and Pompe (53). Specifically, PE is robust to observational or dynamical noise in the data, is generally faster than other time series complexity measures, and avoids restrictive parametric model assumptions (54). PE has been successfully applied to various fields such as economics (54) and mechanical engineering (55). In biomedical studies, it has been used to quantify predictability of infectious diseases (56), to differentiate between epileptic and nonepileptic electroencephalographic recordings (57), as well as to typify the complexity of short heart period variability series (58).

Our analysis revealed disparate patterns of predictability across place categories. We further explored the factors associated with mobility predictability using statistical methods, controlling for a range of covariates and spatiotemporal autocorrelation. Interestingly, we found that the predictability of all place categories was impacted by four groups of factors, namely, weather conditions, population size, COVID-19 growth, and government policies. The nonlinear effects of these factors on mobility predictability were estimated using a generalized additive model (GAM).

## Results

### Reactive mobility changes in place categories

We used data from the Google COVID-19 Community Mobility Reports (59) to track changes in foot traffic to six place categories (residential locations, workplaces, transit stations, parks, groceries and pharmacies, and retail and recreation) from 2020 February 15 to 2021 November 23. We focus on the human mobility during the period before the start of the first Omicron wave. The time series measured the daily percent change from baseline in visitation, where the baseline is defined as the median daily visitor numbers from the 5-week period of 2020 January 3– 2020 February 6 (59). To remove outliers and weekly oscillations (Fig. S1), we performed a data preprocessing to better track the mobility trend over time (Figs. S2–S3). The number of counties analyzed for each day varies, depending on the availability of mobility data. For each category, the minimum and maximum numbers of counties for each day are retail and recreation 570–2,299; groceries and pharmacies 461–2,256; parks 69–589; transit stations 152–937; workplaces 427–2,101; residences 583–1,113.

**Fig. 1.**
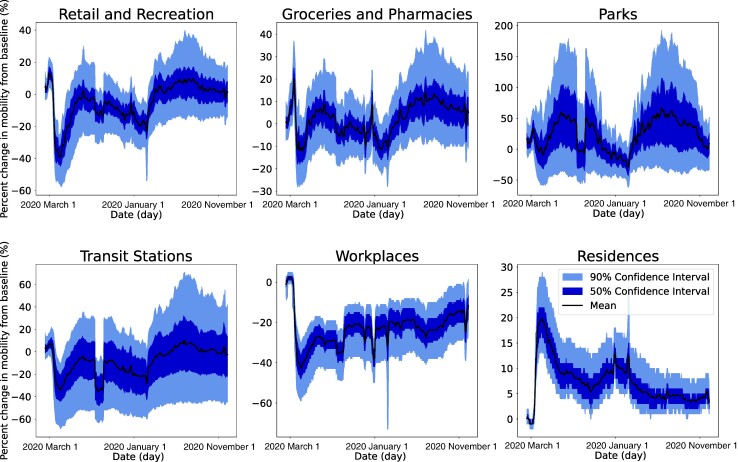
Google COVID-19 community mobility reports data. Time series show the county-level percent change in visitation from baseline across six categories (retail and recreation, groceries and pharmacies, parks, transit stations, workplaces, and residences) in the United States. Solid black lines show the median and shaded areas represent 50 and 90% confidence intervals, respectively. Data were preprocessed to remove outliers and weekly oscillations to better show the mobility trend over time (Supplementary material). The baseline period is defined as the 5-week period 2020 January 3 to 2020 February 6.

Mobility trends for six place categories in the Unites States are presented in Fig. 1. Mobility to residences peaked during the spring wave in 2020, following stay-at-home orders enforced in many jurisdictions across the United States. Subsequently, visitation to residences followed an overall decreasing trend, with another lower peak during the winter of 2020, when COVID-19 surged across the United States. Visits to residences can partially represent the time people staying at home (i.e. when people stay at home for a longer time, there will likely be more recorded visitors to residences). During the periods when COVID-19 cases grew rapidly (e.g. spring 2020 and winter 2020), people may stay at home longer following stay-at-home orders or voluntarily to avoid infection risk in other places. As a result, mobility to residences tends to be above the baseline during these periods.

In contrast to residences, visitation to other place categories dropped precipitously following the strict control measures in March 2020, up to 60% for workplaces and transit stations in certain counties. Mobility to workplaces gradually increased throughout late 2020 and 2021 but did not recover to the baseline level by November 2021. Visitation to transit stations resumed to the baseline level in 2021. Groceries and pharmacies were the least impacted places during the COVID-19 pandemic with the maximal mobility reduction around 15% for the median value. The mobility to groceries and pharmacies, as well as retail and recreation, was able to resume baseline-level mobility in the summer of 2021. Park visitation showed a more regular seasonal cycle—more visitation in summers and less visitation in winters. For all place categories except residences, there was an anomalous drop in the mobility data during the summer of 2020, possibly caused by changes in the data calculation method.

**Fig. 2.**
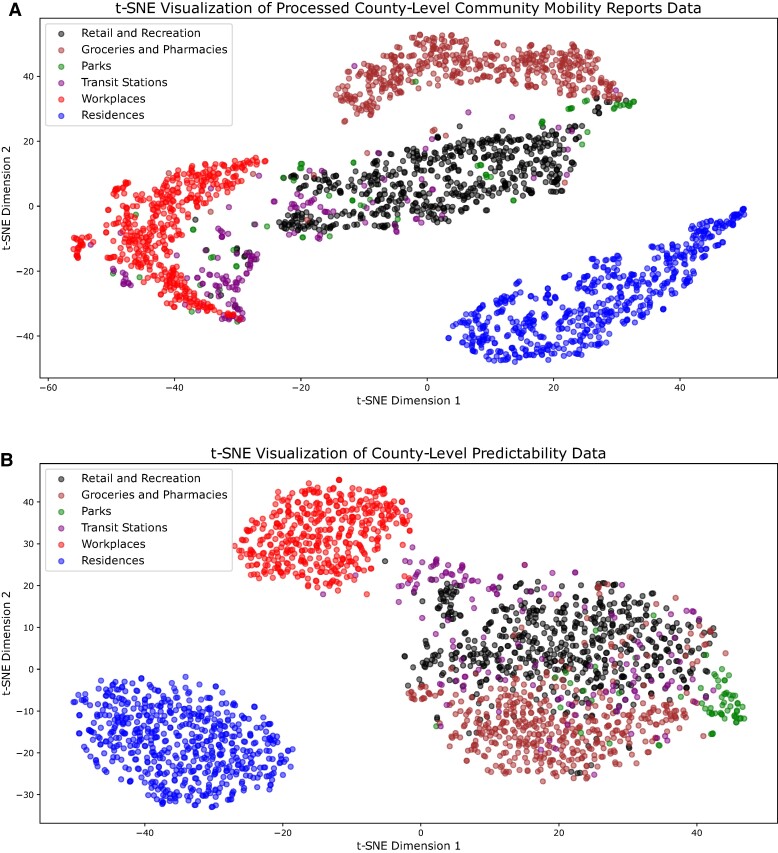
Clustering of mobility patterns across different place categories. We performed t-SNE clustering using the mobility time series (A) and predictability time series (B) at the county level. Each dot represents a time series (mobility in A and predictability in B) for a place category in a single county from 2020 February 15 to 2021 November 23. Colors are used to distinguish different place categories.

Human mobility to different place categories may share similar patterns. To explore this similarity across place categories, we performed a t-Distributed Stochastic Neighbor Embedding (t-SNE) clustering (Materials and methods) using the mobility time series for each place category in US counties with complete mobility data. For workplaces, residences, retail and recreation, and transit stations, mobility time series from different US counties tend to cluster together (Fig. 2A), suggesting that mobility to each of these place categories shared similar patterns across locations. However, these place categories form distant clusters, indicating that they had distinct mobility patterns. Parks and transit stations had different mobility patterns across US counties, as data points for different counties are distant from each other (Fig. 2A).

**Fig. 3.**
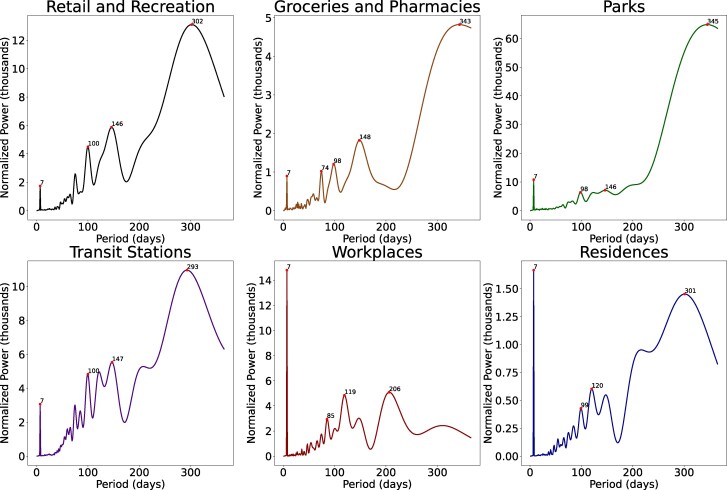
Periodicity of human mobility to different place categories. For each place category, we performed a Fourier analysis on the mobility time series in each county without missing data from 2020 February 15 to 2021 November 23. We computed the power corresponding to different periods from 1 to 365 days and averaged the power across counties. Periods with strong powers within a neighborhood of periods are highlighted for each place category.

To understand the seasonality of mobility, we performed a Fourier analysis to examine the power within mobility time series corresponding to different periods (Materials and methods) (Fig. 3). From 2020 February 15 to 2021 November 23, mobility to workplaces and residences had a strong weekly pattern with a period of 7 days, suggesting that the COVID-19 pandemic have not changed the weekly rhythm of working. Other place categories also showed a weekly periodicity, but the power was relatively lower. Visits to parks were dominated by a period of 345 days, reflecting an annual seasonality. Mobility to retail and recreation, groceries and pharmacies, and transition stations also had a long period around 300 days, possibly due to that the annual seasonality was disrupted by the COVID-19 pandemic.

### Predictability of human mobility

We computed the predictability of human mobility within a time interval using PE. For human mobility, a higher PE implies a more random time series with less predictability. As a result, we defined the predictability of human mobility as $1-h_{n}$, where $h_{n}\in [0,1]$ is the normalized PE (Materials and methods). This definition has been used to quantify the predictability of infectious disease time series (56). For each place category and county, we used a sliding time window of 30 days starting from each day to compute daily PE. The PE value for each day quantifies the predictability of mobility over the succeeding 30-day period, with $h_{n}=1$ representing complete randomness and $h_{u}=0$ representing complete order (i.e. strictly increasing or decreasing). For each day, the minimum and maximum numbers of counties with predictability data are listed below: retail and recreation 569–2,205; groceries and pharmacies 461–2,164; parks 69–552; transit stations 149–879; workplaces 427–2,047; residences 580–1,090.

**Fig. 4.**
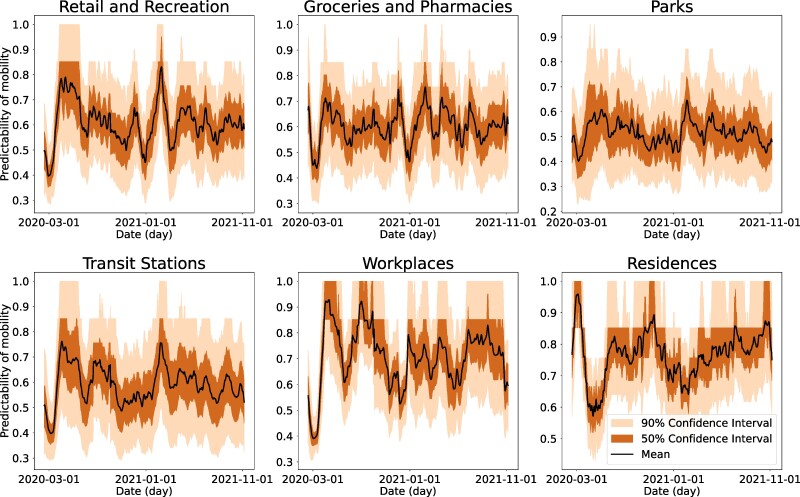
Predictability of mobility in six place categories. For each county, the normalized PE $h_{n}$ was computed for a 30-day sliding time window starting from each day. The figures show the daily predictability, $1-h_{n}$, for each place categories at the county level. The median (solid black lines), 50 and 90% CIs (shaded areas) were calculated using all US counties with mobility data. A predictability of zero represents complete randomness and a predictability of one represents complete order (i.e. strictly increasing or decreasing).

The mobility predictability exhibited differential patterns across place categories (Fig. 4). Most predictability values ranged between 0.4 and 0.8, suggesting a certain level of regularity of mobility in all place categories. Notably, the predictability for residences reached a peak above 0.9 in March 2020, possibly due to synchronized stay-at-home orders effected in many locations in the United States. In contrast, the predictability for other five place categories dropped significantly during the same period, indicating highly irregular mobility patterns under the disruption caused by the pandemic. In May 2020, visitation to places other than residences showed an increased predictability, reflecting a more regular increasing trend of mobility following relaxation of control measures. The predictability for residences fell during the same period—the change of predictability generally followed opposite directions for residences and other place categories. After summer 2020, the predictability for all place categories fluctuated but with a smaller magnitude. Relatively, predictability for workplaces and residences had the most volatile fluctuations as mobility in these places was more prone to be impacted by government policies and voluntary behavioral changes. Mobility predictability for parks and groceries and pharmacies were more stable throughout the study period. We performed a t-SNE clustering to examine the similarity between predictability time series for different place categories in US counties (Fig. 2B). Residences and workplaces formed their own clusters, while other place categories clustered together, sharing similar patterns of predictability change.

**Fig. 5.**
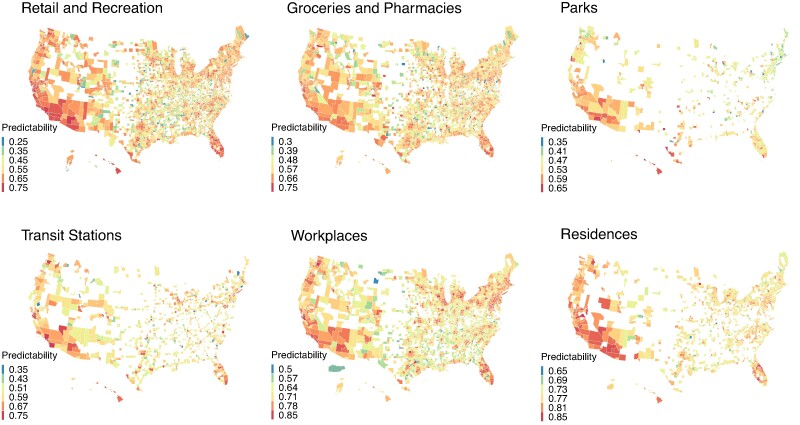
Average mobility predictability at the US county level. For each county and place category, we show the predictability averaged over the period from 2020 February 15 to 2021 November 23. Counties without complete predictability data for each place category were shown as blank. The range of predictability is shown in the color bars.

We visualize the spatial pattern of mobility predictability across US counties using county-level predictability averaged over the study period (Fig. 5). Mobility data for different place categories had varying levels of missingness—a good coverage for retail and recreation and groceries and pharmacies versus a limited coverage for parks and transit stations. Despite the data missingness, we can identify several spatial patterns for mobility predictability. For instance, predictability for residences and workplaces was higher in the west coast and Florida. For retail and recreation and groceries and pharmacies, several states such as California, Arizona, and Florida had a higher predictability than other locations in the middle of the United States. Visitation to parks was least predictable in the northeast United States. Mobility to transit stations was more predictable in metropolitan areas such as San Francisco, Seattle, Los Angeles, San Diego, etc., where commuters more relied on public transportation systems.

To better compare mobility predictability across the United States, we show the distribution of average mobility predictability for each place category in the counties within each of the four US regions defined by the US Census Bureau—Northeast, South, Midwest, and West (Fig. S4). In general, mobility in the West region was more predictability, especially for retail and recreation, groceries and pharmacies, parks, and residences. Within each region, there was a large variation of mobility predictability across counties. Notably, the predictability for visiting parks exhibited the strongest variation across the four US regions among the examined six place categories.

### Factors associated with mobility predictability

A myriad of factors can impact human mobility. We employed a GAM to estimate the effects of a range of factors on mobility predictability (Materials and methods). To reduce the temporal autocorrelation in predictability data, we used the *monthly* predictability computed using nonoverlapping mobility data for each month separately as response variables. We controlled for a range of covariates at the county level including demographic and socioeconomic factors, disease statistics, commuting behavior, governmental response, and weather conditions (Materials and methods). Specifically, we included the following covariates at the US county level: (i) log-transformed population size; (ii) percentage of Black residents; (iii) percentage of Hispanic residents; (iv) median household income; (v) monthly new COVID-19 cases; (vi) a binary indicator for increasing COVID-19 cases; (vii) percentage of residents over 60 years old; (viii) percentage of residents over 25 years old without high school diploma; (ix) the stringency of government responses to the COVID-19 pandemic, as measured by the Oxford government stringency index; (x) monthly average temperature; (xi) monthly precipitation; (xii) percentage of residents commuting to work by public transit; (xiii) percentage of residents working from home. A correlation analysis was performed to ensure that no strong collinearity existed in the covariates (Fig. S5). We used tensor product smooth terms to account for the spatial autocorrelation in the response variables and potential nonlinearities and interactions between some variables. A residual analysis was performed to ensure no spatial and temporal autocorrelations existed in the residuals (Figs. S6–S9).

To quantify the impacts of covariates on mobility predictability, we ranked the covariates using *F*-values for each place category and selected the top five most impactful factors. Interestingly, this analysis identified four groups of variables that persistently imposed strong impacts on mobility predictability across all place categories (Table S1). These include weather conditions (monthly precipitation and temperature), demographic and socioeconomic factors (population size), local COVID-19 spread (increasing or decreasing cases), and government policy mandates (the Oxford government stringency index).

**Fig. 6.**
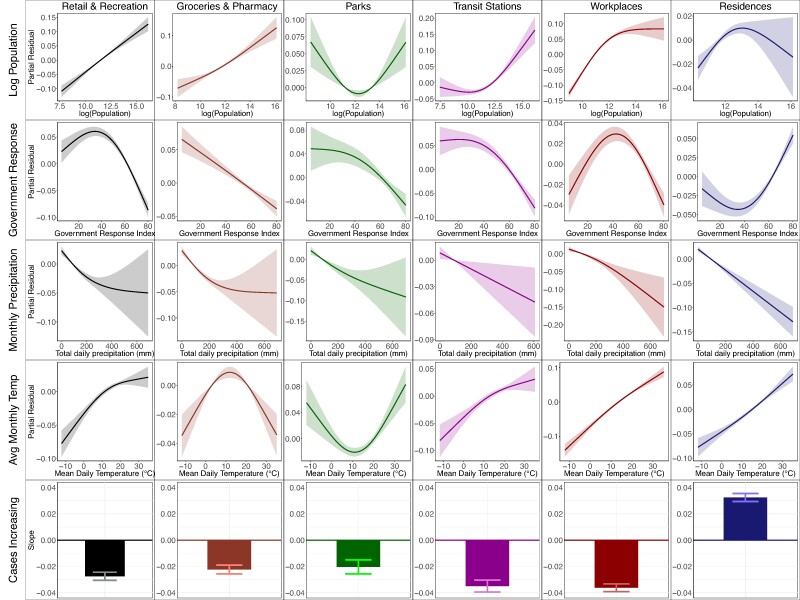
The partial effects of population size, government response, precipitation, temperature, and infection trend on mobility predictability for six place categories. Statistical analysis was performed using a GAM controlling for a variety of covariates at the county level. The solid lines show mean values, and the shaded areas represent 95% CIs. For the binary variable, case increasing, we show the slope coefficients representing the change of predictability when the variable changes from 0 to 1. The bars show the 95% CIs. Partial effects were estimated for the period from 2020 February 15 to 2021 November 23, using all counties with monthly mobility predictability data.

We visualize the partial effects of several factors on mobility predictability for each place category (Fig. 6, Fig. S6). (i) Weather conditions had profound impacts on human mobility. A higher monthly precipitation was associated with a lower mobility predictability for all place categories, indicating an overall more irregular mobility pattern during months with more rains. Temperature was positively associated with mobility predictability for residences, workplaces, retail and recreation, and transit stations, but had a nonlinear impact on predictability for groceries and pharmacies and parks. (ii). Population size was positively associated with mobility predictability except for residences and parks. Generally, mobility in counties with more population tended to be more predictable. (iii). Local infection trend also had strong impacts on predictability. An increasing trend of COVID-19 cases was associated with a higher predictability for residences but a lower predictability for other place categories. (iv) The impact of government response index was more subtle. Stronger government interventions reduced predictability for mobility in groceries and pharmacies, transit stations, and parks. Intermediate level of government interventions increased predictability for workplaces and retail and recreation and rendered mobility in residences less predictable, showing nonlinear effects for these place categories.

## Discussion

Understanding whether human mobility can be predicted during a public health emergency is critical for epidemic modeling and disease control. In this study, using an information-theoretic metric and mobility data from Google COVID-19 Community Mobility Reports, we quantified the predictability of mobility in six place categories at the US county level. We found differential patterns of predictability between residences and other place categories and revealed the spatial variations in mobility predictability. We further used statistical methods to identify common key factors that impacted predictability and quantified their effects for different place categories.

Our analysis found that mobility in the six place categories had different patterns of predictability. Notably, predictability change in residences was mostly in the opposite direction with other places. In addition, government policies had disparate impacts on different places. As shown in Fig. 1, visitation to workplaces and residences was more impacted by NPIs but not so much for groceries and pharmacies and parks. Indeed, essential activities such as getting foods and medicines should be less disrupted by government policies. This finding highlights the need to reduce infection risk in the activities that are not easily altered by administrative orders during future public health emergencies (e.g. improving ventilation and providing food delivery).

The predictability of human mobility varied across the US geographically. While some US states shared similar predictability patterns in certain place categories, the underlying mechanism may be different. For instance, on average, both the west coast and Florida had high predictability for visits to residences. Major cities on the west coast (e.g. San Francisco and Seattle) were generally more vigilant to COVID-19 and have implemented relatively strict NPIs. The high predictability might be due to the synchronized mobility under stay-at-home orders. In contrast, Florida has implemented minimal NPIs, which might also lead to more predictable mobility as people's routine activities were less disrupted. As another example, mobility to workplaces in the west coast was more predictable, possibly because there are several major metropolitan areas in these regions with a large number of commuters, whose mobility to workplaces was strongly modulated by NPIs. Whereas, visiting to workplaces in Florida was more predictable potentially because work-related mobility was less impacted by NPIs. The absence of strong NPIs might explain why Florida stood out with high predictability in several place categories; however, the actual reasons need to be further studied.

After the removal of most governmental interventions, mobility predictability still fluctuated throughout the study period. Such fluctuations were more attributable to voluntary behavior changes in response to infection risk. Understanding the reactive behavior changes in different communities and the underlying mechanisms can help design improved interventions to better incentivize people to change their behaviors to curb disease spread.

Mobility data derived mobile phones have been used to study mobility predictability after natural disasters. For instance, Lu et al. (60, 61) analyzed the predictability of population displacement after the 2010 Haiti earthquake. Contrary to the common belief that human mobility becomes less predictable after the disaster, they found the predictability of people's trajectories remained high during the 3-month period after the earthquake. Our finding of the relatively high mobility predictability during the COVID-19 pandemic in US counties agrees with this previous result.

In this study, we used PE to measure the randomness of aggregated mobility time series. Since the aggregated time series were synthesized from a large number of individuals with varying socioeconomic and demographic characteristics, our study has the limitation that the findings cannot reflect the change of predictability among different groups of populations during the COVID-19 pandemic. More granular mobility data with associated socioeconomic and demographic information are needed to support in-depth analyzes in future works. Another limitation of the study arises from the Google COVID-19 Community Mobility Reports data. The mobility data used the median value of visitors to different place categories from the 5-week period (2020 January 3—February 6) as the baseline, which only reflects human mobility during the wintertime. Since the mobility data did not cover the period before 2020, our analysis cannot elucidate the overall mobility change caused by COVID-19 relative to the same dates in previous years.

Real-time forecasting of human mobility has broad applications in disease control and public health. Our findings indicate that human mobility during public health emergencies had a certain level of predictability, providing a theoretical basis for forecasting reactive behavior changes. However, we discovered strong temporal variations in mobility predictability, indicating a pronounced nonstationary nature of the mobility dynamics. This nonstationarity imposes a challenge in operational mobility forecasting in real time. For instance, models trained using historical data may not produce reliable forecasts for the future. Existing predictive models using human mobility data typically assume constant mobility or predefined mobility change in the forecast horizon. Those assumptions may generate suboptimal predictions in locations with low predictability of mobility. While our analysis did not aim to find which mobility metrics should be employed in disease forecasting, we identified several factors associated with lower mobility predictability. For example, mobility in locations with small populations and high precipitation was generally less predictable during the COVID-19 pandemic, which may lead to larger uncertainty in disease dynamics and create challenges in real-time forecasting. Further analyzes on the choice of mobility metrics and their utilities in disease forecasting across different locations are warranted in future research.

## Materials and methods

### Data

We used data from the Google COVID-19 Community Mobility Reports (59), downloaded from Harvard Dataverse (62). This US county-level dataset detailed the daily percent change from baseline in visitation to six different categories—parks, groceries and pharmacies, retail and recreation, transit stations, workplaces, and residences. The baseline is the median value from the 5-week period 2020 January 3 to February 6 (59). We limited our analysis to data from 2020 February 15 to 2021 November 23, prior to the wide spread of the Omicron variant. Before running the analysis, we performed preprocessing to remove outliers and deal with data missingness in the raw data. Details are presented in Supplementary material. Demographic and socioeconomic statistics at the county level were obtained from the American Community Survey data in 2021 (63), downloaded using the R package tidycensus (64). COVID-19 data were downloaded from the Johns Hopkins Coronavirus Resource Center (65). Temperature and precipitation data were compiled from the North America Land Data Assimilation System Project (66). COVID-19 government response index was downloaded from the University of Oxford COVID-19 Government Response Tracker (67).

### Permutation entropy

The PE method has three inputs: $\{x_{t}{\}}_{t=1,\cdots ,T}$, the time series with length *T*; *τ*—the embedding time delay; and *n*—the embedding dimension or order (53). In our analysis, we fixed τ=1, as in previous studies using PE (53). The output is a single number, representing the complexity of the time series *x*. Intuitively, PE uses the order type of subsequences of the time series to quantify the complexity. As it only considers the relative order of two consecutive numbers (instead of using the exact numerical values), it bypasses the need to arbitrarily define data bins for continuous variables for Shannon entropy and is not impacted by the scale of data. PE is also more robust to noises in the time series data.

For a time series $\{x_{t}{\}}_{t=1,\cdots ,T}$, we examine all n! permutations *π* of order *n* which are considered as possible order types of *n* different numbers. For each *π*, we compute the relative frequency


$$p(\pi )=\frac{\#\;\{t|t\leq T-n,\;(x_{t+1},\cdots ,x_{t+n})\;\mathrm{has}\;\mathrm{type}\;\pi \}}{T-n+1}.$$

The PE of order n≥2 is defined as


H(n)=−∑p(π)logp(π),

where the sum runs over all n! permutations *π* of order *n*. H(n) ranges from 0 to log(n!). A strictly increasing or decreasing time series will have PE of 0 and a completely random time series will have PE of log(n!). We can normalize the PE by dividing it by log(n!):


$$h_{n}=H(n)/\log\;(n!).$$

Here $h_{n}$ is normalized to ensure the range to be within 0 and 1. In our analysis, we used $1-h_{n}$ to define the predictability of mobility.

To compute $h_{n}$, we need to define the embedding time delay *τ* and the order *n*. A large embedding dimension means that we can detect more distinct ordinal patterns. However, there will be fewer observations of these ordinal patterns, causing our estimates p(π) to be less reliable (68). Bandt and Pompe (53) provided three heuristics for choosing effective values of *τ* and *n*. Namely, τ=1, n≤7, and T>n!. Staniek and Lehnertz (69) provided empirical evidence for the usefulness of these heuristics in calculating PE. In our analysis, we set τ=1. As we used a 30-day time window (T=30), we tested n=2,3,4 and found that n=4 in general led to smallest $h_{n}$ values. We therefore set n=4 in our analysis to estimate the upper limit of predictability across different orders. For each county in each category, we calculated the predictability of the processed data in sliding 30-day time windows. We used the ordpy.PE function in Python with an embedding dimension of 4 and embedding time delay of 1. If the 30-day time series had more than three missing data points, we replaced the predictability with a NaN value.

### Statistical analysis

We performed a t-SNE clustering analysis to examine the similarity between different mobility time series. t-SNE is a nonlinear dimensionality reduction technique for embedding high-dimensional data for visualization in a low-dimensional space of two or three dimensions (70, 71). In the analysis, the mobility data were standardized to have zero mean and unit variance, including only counties with no missing data. The standardized data were then reduced to two dimensions using t-SNE from the sklearn library in Python (72). t-SNE reduces high-dimensional data to two dimensions by constructing probability distributions of pairwise similarities in both the high-dimensional and low-dimensional spaces. It then iteratively adjusts the low-dimensional points to minimize the Kullback–Leibler divergence between these distributions, preserving the local structure and clustering of the original data.

A Fourier analysis was employed to examine the seasonality of human mobility using data from 2020 February 15 to 2021 November 23. For the mobility time series in each county without missing data, we performed a Fourier transform to convert the time series from its original domain to a representation in the frequency domain corresponding to different periods. Before running the analysis, we subtracted the mean from the time series in each county and normalized the Fourier transform by dividing it by the length of the time series. This normalization technique allows for comparison between signals with different sampling rates. We computed the power within the time series corresponding to different periods from 1 to 365 days with a 1-day interval and averaged the power across counties. The periods with locally maximum powers are highlighted in Fig. 3.

We used a GAM model to estimate the effect of factors on mobility predictability. GAM is effective in handling data with spatiotemporal autocorrelations and is computationally efficient. Denote $P_{i,t}$ as the predictability for each place category in county *i* and month *t*. The model can be expressed as:


$$\begin{array}{rl} P_{i,t} & =\alpha +\mathrm{te}(\mathrm{latitud}e_{i},\;\mathrm{longitud}e_{i},t)+s(\mathrm{latitud}e_{i},\;\mathrm{longitud}e_{i},\mathrm{bs}="\mathrm{tp}")\\ & \;+\beta_{1}(\log\;\mathrm{populatio}n_{i})+\beta_{2}(\mathrm{percent}\;\mathrm{Black}\;\mathrm{resident}s_{i})\\ & \;+\beta_{3}(\mathrm{percent}\;\mathrm{Hispanic}\;\mathrm{resident}s_{i})\\ & \;+\beta_{4}(\mathrm{median}\;\mathrm{household}\;\mathrm{incom}e_{i})+\beta_{5}(\mathrm{monthly}\;\mathrm{case}s_{i,t})\\ & \;+\beta_{6}(\mathrm{case}\;\mathrm{increasin}g_{i,t})+\beta_{7}(\mathrm{percent}\;\mathrm{population}\;\mathrm{over}\;60_{i})\\ & \;+\beta_{8}(\mathrm{percent}\;\mathrm{residents}\;\mathrm{over}\;25\;\mathrm{without}\;\mathrm{high}\;\mathrm{school}\;\mathrm{diplom}a_{i})\\ & \;+\beta_{9}(\mathrm{Oxford}\;\mathrm{government}\;\mathrm{response}\;\mathrm{index})\\ & \;+\beta_{10}(\mathrm{monthly}\;\mathrm{temperatur}e_{i,t})+\beta_{11}(\mathrm{monthly}\;\mathrm{precipitatio}n_{i,t})\\ & \;+\beta_{12}(\mathrm{percent}\;\mathrm{residents}\;\mathrm{commuting}\;\mathrm{to}\;\mathrm{work}\;\mathrm{by}\;\mathrm{public}\;\mathrm{transi}t_{i})\\ & \;+\beta_{13}(\mathrm{percent}\;\mathrm{residents}\;\mathrm{working}\;\mathrm{from}\;\mathrm{hom}e_{i}). \end{array}$$

Here, *α* is the intercept. Case increasing is a binary variable representing the trend of infection in each county. For each month and each county, we performed a linear regression, y=kx+b, where *x* is the day since the beginning of the time series and *y* is the daily confirmed cases. The linear regression was performed for the daily confirmed cases with each month. Case increasing was defined as 1 if the slope of the regression line, *k*, is positive and 0 otherwise. Government response index was at the state level, and we used the monthly average in each state to represent control stringency in all counties within that state. Percent residents commuting to work by public transit and percent residents working from home represent commuting behavior of residents in each county. We included them in the GAM to control for the impact of commuting behavior on mobility predictability. We accounted for spatial autocorrelation by including the term $te(\mathrm{latitud}e_{i},\;\mathrm{longitud}e_{i},t)$. This tensor product smooth term allows the model to account for nonlinear relationships between the response variable and the spatial coordinates, latitude, and longitude, calculated to be the geographical center of their respective counties. This tensor product term is a smooth function of several variables where the basis is defined using tensor products of bases for single variables (here the variables are latitude, longitude, and time). An addition term $s(\mathrm{latitud}e_{i},\;\mathrm{longitud}e_{i},\mathrm{bs}="\mathrm{tp}")$ was used, where bs="tp" represents a thin plate spline that is used primarily for smoothing over two or more dimensions. This term was used to additionally capture spatial patterns that are not necessarily time-dependent to further reduce spatial autocorrelation. The basis dimension for the smooth terms was set to be 3, chosen in order to minimize the Akaike Information Criterion, prevent overfitting in the response curves, and yield reasonable results with the gam.check diagnostics tool. The GAM model was implemented using the mgcv package in R (73).

We plotted the correlations between all pairs of covariates to ensure that no strong collinearity existed in the explanatory variables (Fig. S5). Data from all county–month combinations with available predictability data were used in the analysis. To ensure the GAM captured the spatiotemporal autocorrelation in the response variable, we performed a residual analysis. Statistical tests indicated that there were no spatial (Moran's I test) (Fig. S8) and temporal (Durbin–Watson test) autocorrelations in the residuals (Fig. S9). Details on statistical tests are provided in Supplementary material.

## Supplementary Material

pgae308_Supplementary_Data

## Data Availability

All data used in this study are publicly available. The Google COVID-19 Community Mobility Reports data were downloaded from Harvard Dataverse. Demographic and socioeconomic statistics were obtained from the American Community Survey. COVID-19 data were downloaded from the Johns Hopkins Coronavirus Resource Center. Temperature and precipitation data were compiled from the North America Land Data Assimilation System Project. COVID-19 government response index was downloaded from the University of Oxford COVID-19 Government Response Tracker. All the data and custom code supporting the statistical analysis are publicly available at GitHub (https://github.com/cheatingthemichal/USCOVID-Mobility-Predictability).
