# Supplementary material for: Predictability of human mobility during the COVID-19 pandemic in the United States
Source: PNAS Nexus. 2024 Jul 26;3(8):pgae308. doi: 10.1093/pnasnexus/pgae308 (PMC11305134; doi:10.1093/pnasnexus/pgae308)
Supplement: pgae308_Supplementary_Data [file pgae308_supplementary_data.docx]

**
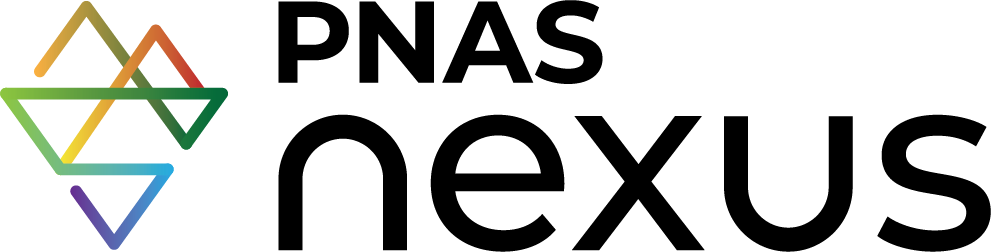
**

**Supplementary Information for**

Predictability of human mobility during the COVID-19 pandemic in the United States

Michal Hajlasz^1^, Sen Pei^2^

1. Department of Computer Science, Columbia University, 10027 New York, USA

2. Department of Environmental Health Sciences, Mailman School of Public Health, Columbia University, 10032 New York, USA

*Corresponding author: Sen Pei.

**Email:** sp3449@cumc.columbia.edu

**This PDF file includes:**

Supplementary text

Figures S1 to S9

Tables S1

SI References

**Supplementary Information Text**

**Data preprocessing**

The raw Google COVID-19 Community Mobility Reports data have many outliers, as well as weekly oscillations (Fig. S1). Because these weekly oscillations are not indicative of any long-term trends, they create noise when calculating predictability. We preprocessed the data in order to remove these outliers and oscillations. We calculated the median of the 7-day window around each point in each county time series in each category.

Many of the counties have missing data for a large percentage of days. In Fig. S2, we graph the number of counties in each category whose missingness is within a percentage range (0-20%, 20%-40%, 40%-60%, 60%-80%, and 80%-100%). As we can see, in every category, there are many counties with over 80% data missingness, except for workplaces. Over 2,000 counties have less than 20% missingness for workplaces (red bar), and over 2,000 counties have more than 80% missingness for parks (green bar on the right). This indicates that workplaces have better coverage at the county level than parks and transit stations. We tested how limiting our regression to only those counties with missingness under a certain percentage impacted the performance of our model. This helped us balance the need for a larger dataset with the goal of ensuring data quality.

In Fig. S3, we illustrate the percent change in Retail and Recreation visitation from baseline in Lawrence County, AL. The raw data, presented in blue, is only complete for the first two months. Beyond this, data points become sparse, with a total data availability of 32.36%. To address this data missingness, we adopted two approaches. In the first approach, depicted in yellow (the yellow curve is completely covered by the red curve that represents the overlapped smooth curves obtained using np.median and np.nanmedian), we used np.median when removing outliers in the sliding window algorithm described above. This has the effect of removing data points if missing values are present in their respective 7-day window. This yields a conservative trend line that strictly adheres to the non-missing data. In the second approach, depicted in green, we use np.nanmedian when removing outliers in our sliding window algorithm. This effectively infers missing values based on the non-missing values within their respective 7-day window. This results in a more complete time series for the counties with limited data availability. However, when data is too sparse, it can be difficult to accurately estimate order type frequency, which is necessary for reliable predictability calculations. As Fig. S3 demonstrates, the two approaches yield overlapping data (in red) in absence of missingness, while only the np.nanmedian approach (in green) yields data in instances of sparse raw data. In the final model, we used the np.median function in the sliding window algorithm, which yielded higher R^2^ values than the np.nanmedian function.

In Fig. 1, we graph the daily processed percent change from baseline in visitation for each category. Date is on the x-axis and percent change is on the y-axis. The percent change is smoothed with our sliding-window algorithm. As we can see, after data preprocessing, the average time series has less local and global outliers, causing long-term trends to be more visible.

**Permutation entropy**

We show how permutation entropy is calculated using definitions and examples provided in Bandt and Pompe (1). Let $x=\{4,7,9,10,6,11,3\}$, $n=2$, and $\tau=1$. We first look at all consecutive neighbors of length $n$ in $x$. In this example, we have pairs $(4,7)$, $\left( 7,9 \right)$, $(9,10)$, $(10,6)$, $(6,11)$ and $(11,3$). $\tau$ is the distance between each element of our tuples. For example, if $\tau=2$, we would have pairs $(4,9)$, $(7,10)$, $(9,6)$, $(10,11)$, and $(6,3)$. Next, we look at the order type of each tuple. Here $(7, 9)$ has order type $\langle0, 1\rangle$ because $7<9$. $(10, 6)$ has order type $\langle1, 0\rangle$ because $10>6$. Ties in values can be disrupted with small amounts of Gaussian noise. We have 4 tuples of order type $\langle0,1\rangle$ and 2 tuples of order type $\langle1,0\rangle$. In total, we have $T-n+1=6$ tuples.

We can now calculate $p(\pi)$. Our best estimate of the frequency of order type $\pi$ occurring in a finite sequence. In our example, $p\left( \left\langle0,1 \right\rangle\right)=4/6$ and $p\left( \left\langle1,0 \right\rangle\right)=2/6$. More generally,

$$p\left( \pi\right)=\frac{\#\{t|t\leq T-n, \left( x_{t+1},\cdots,x_{t+n} \right) has type \pi\}}{T-n+1}.$$

Finally, we can calculate the permutation entropy $H(n)$ as the Shannon entropy

$$H\left( n \right)=-\sum p\left( \pi\right)\log p\left( \pi\right)$$

summed over all permutations of $\pi$ that appear in our tuples. In our example

$$H\left( 2 \right)=-\frac{4}{6}\log\frac{4}{6}-\frac{2}{6}\log\frac{2}{6}\approx0.918.$$

$H(n)$ ranges from 0 to $\log(n!)$. A strictly increasing or decreasing time series will have permutation entropy of 0 and a completely random time series will have permutation entropy of $\log(n!)$. We can normalize the permutation entropy by dividing it by $\log(n!)$.

$$h_{n}=H(n)/\log(n!).$$

**Residual analysis**

We evaluated the spatial-temporal autocorrelation in the model residuals to confirm that spatial and temporal structures had been annihilated. Specifically, we examined the spatial autocorrelation in residuals from the six place categories using Moran’s I(2, 3). The spatial autocorrelation of the residuals was absent for most months (𝑝 > 0.05) (Fig. S8). Moran’s I is a measure of spatial autocorrelation that characterizes the correlation in a signal among nearby locations in space. Specifically, it is defined as

$$I=\frac{N}{W}\frac{\sum_{i=1}^{N} \sum_{j=1}^{N} w_{ij}(x_{i}-\bar{x})(x_{j}-\bar{x})}{\sum_{i=1}^{N} \left( x_{i}-\bar{x} \right)^{2}},$$

Where $N$ is the number of spatial units, $x$ is the variable of interest (here, the residual for mobility predictability), $\bar{x}$ is the mean of $x$, $w_{ij}$ is a matrix of spatial weights with zeros on the diagonal ($w_{ii}=0$), and 𝑊 is the sum of all $w_{ij}$ ($W=\sum_{i=1}^{N} \sum_{j=1}^{N} w_{ij}$). Here, we define $w_{ij}$ as the adjacency matrix for all ZIP code areas, i.e., $w_{ij}=1$ if locations 𝑖 and 𝑗 are contiguous, $w_{ij}=0$ otherwise. Under the null hypothesis of no autocorrelation, the expected value of 𝐼 is $E(I) = -1/(N - 1)$, which approaches zero as 𝑁 approaches infinity. The p-value of the Moran’s I test is obtained by comparing the computed 𝐼 value with the null distribution.

For each place category, we evaluated the temporal autocorrelation in the residuals using the Durbin-Watson test(4). Results indicate that temporal autocorrelation was not significant in most counties for all place categories (𝑝 > 0.05) (Fig. S9). Durbin-Watson test is used to detect the presence of autocorrelation at lag 1 in the residuals from a regression model. Define $e_{t}$ as the residual at time 𝑡. The Durbin-Watson test statistics is

$$d=\frac{\sum_{t=2}^{T} \left( e_{t}-e_{t-1} \right)^{2}}{\sum_{t=1}^{T} e_{t}^{2}}.$$

Here 𝑇 is the number of observations. The value of 𝑑 ranges between 0 and 4. For large 𝑇, 𝑑 is approximately equal to 2(1 − 𝜌), where 𝜌 is the sample autocorrelation of the residuals. 𝑑 = 2 indicates no autocorrelation; 𝑑 < 2 indicates a positive serial correlation; 𝑑 > 2 indicates a negative serial correlation.

**Fig. S1.** Raw Google COVID-19 Community Mobility Reports data. We graph the daily percent change from baseline in visitation for each category, averaged across all counties. Date is on the x-axis and percent change is on the y-axis. Solid black lines show median values and shaded areas represent 50% and 90% confidence intervals.

**Fig. S2.** Data missingness. Number of counties in each category whose missingness is within a given percentage range.


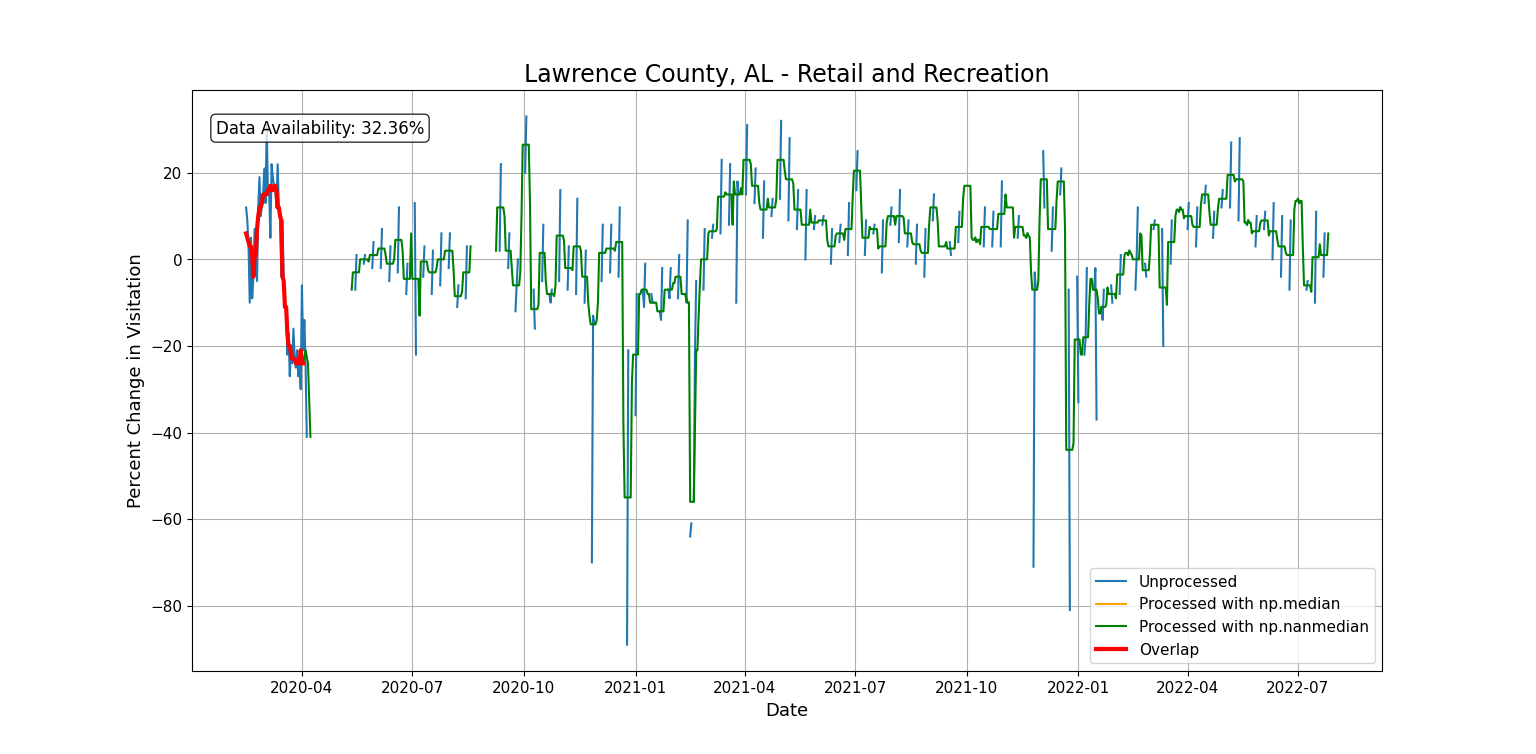
**Fig. S3.** Percent change in Retail and Recreation visitation from baseline in Lawrence County, AL before and after data preprocessing. The blue line shows the original mobility data without preprocessing. The green line shows the smoothed curve using the function np.nanmedian, which calculates median after removing missing values in the 7-day window. Yellow line shows smoothed curve using the function np.median to calculate the median of the 7-day window around each point. If there is a missing value in the 7-day window, the median is set as nan. Because this curve partially overlaps with the curve produced using np.nanmedian (For time series without missing values, np.median and np.nanmedian produce exactly the same smoothed curve), the yellow line is completely covered by the red line, which represents the overlapped curve produced using np.median and np.nanmedian.

**Fig. S4.** Distributions of average mobility predictability in four US regions. For each place category, we show the distribution of mobility predictability in the counties within each of the four US regions defined by the US Census Bureau – Northeast, South, Midwest, and West. Boxes and whiskers show the median, interquartile (IQR), and the range within 1.5 IQR from the 25% and 75% percentiles.


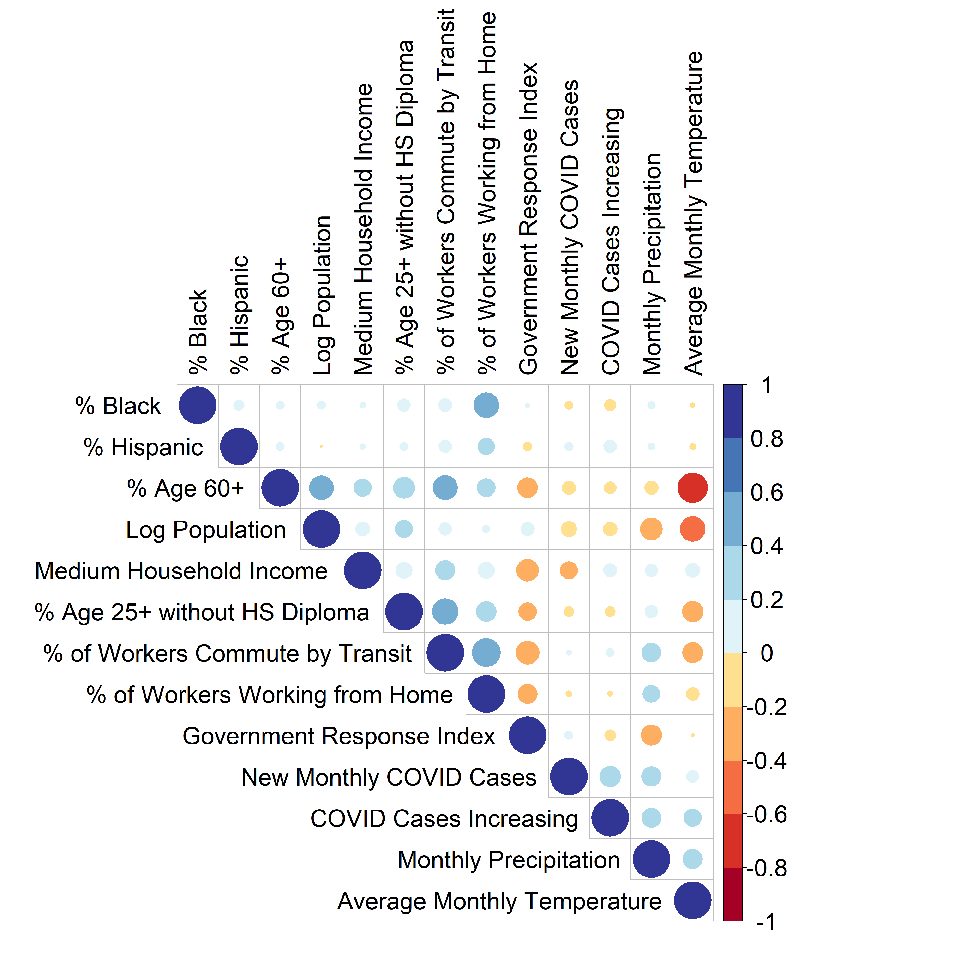


**Fig. S5.** Spearman correlation coefficients between covariates in the statistical model. The size of the disk represents the absolute value of the Spearman correlation coefficient (i.e., a large size corresponds to a stronger correlation). The color of the disk represents the direction of correlation (blue represents positive and red represents negative).


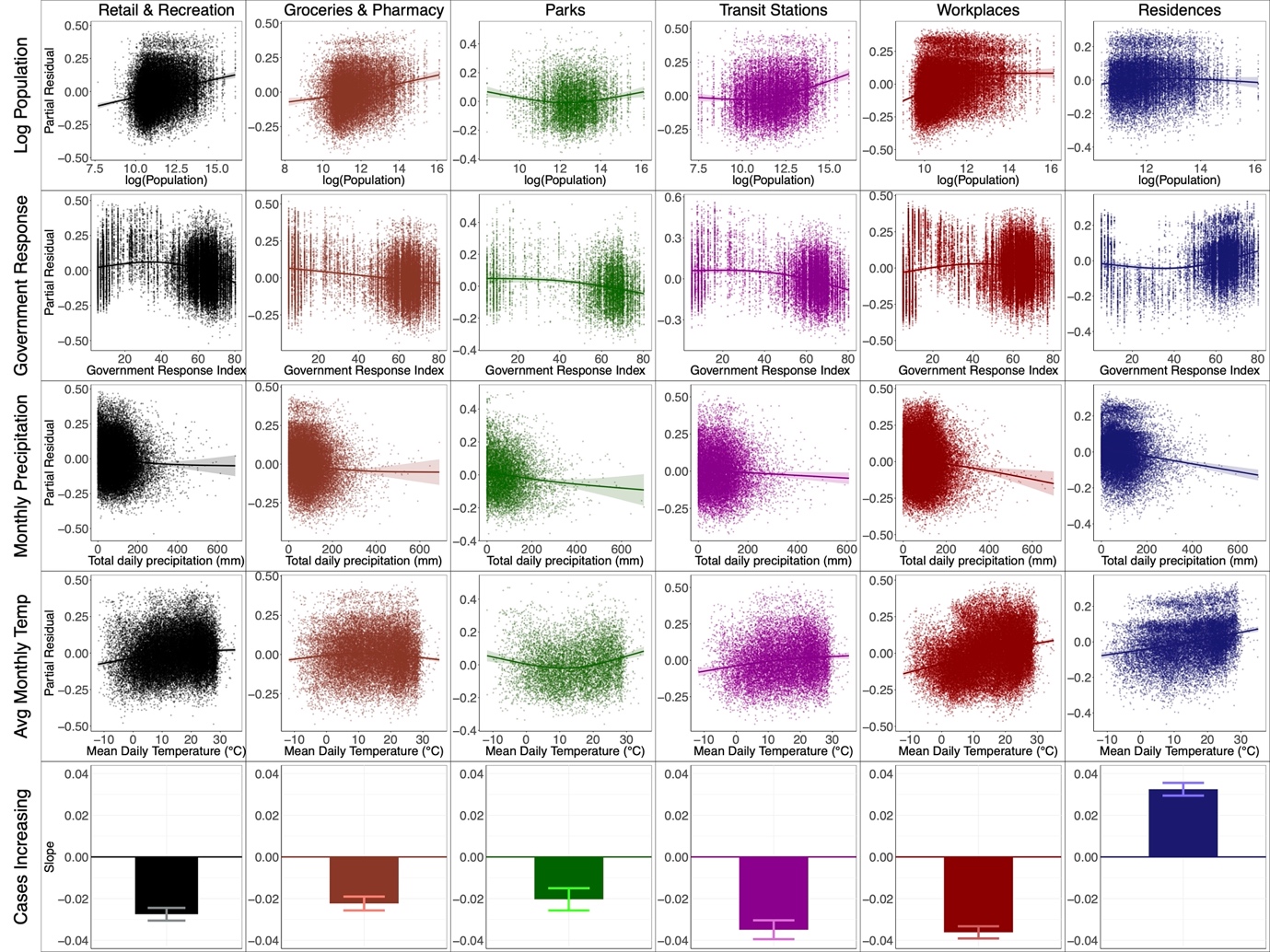


**Fig. S6.** The partial effects of population size, government response, precipitation, temperature, and infection trend on mobility predictability for six place categories. We show partial residuals using points in the plots.


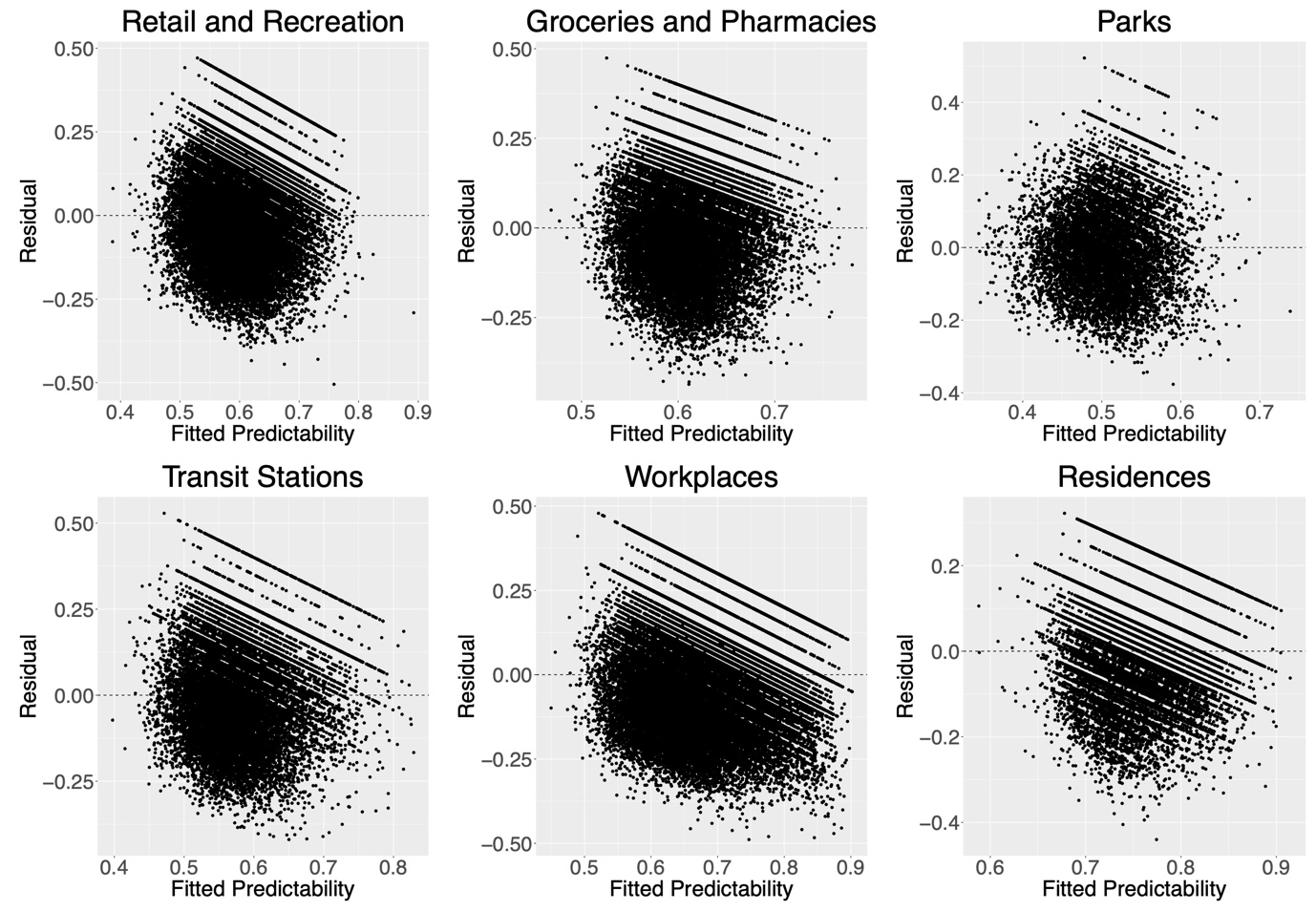


**Fig. S7.** Residuals in the statistical model fitting for six place categories. The response variable, mobility predictability, is shown on the x-axis. The residuals corresponding to different predictability values are shown on the y-axis. The straight lines at higher residual values are probably due to the discrete values of predictability, as can be seen in Fig. 4. In the calculation of PE, certain time series have small denominators in $p(\pi)$, which leads to discrete values of predictability.

**Fig. S8.** Moran’s I test for residual spatial autocorrelation performed for each place category. Within each place category, Moran’s I test was performed for each month. Distributions of p-values for all months are shown. Boxes show the median and interquartile for p-values. The lower whisker represents the smallest value within 1.5$\times$IQR below the lower quartile. The upper whisker represents the largest value within 1.5$\times$IQR above the upper quartile (where the IQR is the middle 50% of the data, and the lower and upper quartiles represent the 25th and 75th percentiles of the data, respectively). The Moran’s I test was two-sided and no adjustment was made for multiple comparisons.

**Fig. S9.** Durbin-Watson test for residual temporal autocorrelation performed for each place category. Distributions of p-values for all counties are shown. Boxes show the median and interquartile for p-values. The lower whisker represents the smallest value within 1.5$\times$IQR below the lower quartile. The upper whisker represents the largest value within 1.5$\times$IQR above the upper quartile (where the IQR is the middle 50% of the data, and the lower and upper quartiles represent the 25th and 75th percentiles of the data, respectively). The Durbin-Watson test was two-sided and no adjustment was made for multiple comparisons.

**Table S1.** Key factors with strong effects on mobility predictability for six place categories. We ranked variables using F-values in the GAM model and present the top 5 variables for each place category.

| **Place category** | **Variable** | **F-values** | **Rank** |
| --- | --- | --- | --- |
| **Groceries and pharmacies** | Log population | 251 | 1 |
|  | Case increasing | 173 | 2 |
|  | Monthly precipitation | 163 | 3 |
|  | Government response index | 100 | 4 |
|  | Monthly temperature | 44 | 5 |
| **Parks** | Monthly temperature | 79 | 1 |
|  | Case increasing | 56 | 2 |
|  | Monthly precipitation | 53 | 3 |
|  | Government response index | 48 | 4 |
|  | Monthly cases | 30 | 5 |
| **Residences** | Case increasing | 433 | 1 |
|  | Monthly temperature | 381 | 2 |
|  | Government response index | 198 | 3 |
|  | Monthly precipitation | 140 | 4 |
|  | Log population | 43 | 5 |
| **Retail and recreation** | Government response index | 427 | 1 |
|  | Log population | 386 | 2 |
|  | Case increasing | 310 | 3 |
|  | Monthly temperature | 157 | 4 |
|  | Monthly precipitation | 117 | 5 |
| **Transit stations** | Case increasing | 229 | 1 |
|  | Government response index | 166 | 2 |
|  | Log population | 147 | 3 |
|  | Monthly temperature | 87 | 4 |
|  | Percent works commuting by public transit | 79 | 5 |
| **Workplaces** | Log population | 1222 | 1 |
|  | Monthly temperature | 881 | 2 |
|  | Case increasing | 590 | 3 |
|  | Government response index | 158 | 4 |
|  | Monthly precipitation | 88 | 5 |

**SI References**

1. C. Bandt, B. Pompe, Permutation Entropy: A Natural Complexity Measure for Time Series. *Phys. Rev. Lett.* **88**, 174102 (2002).

2. P. A. P. Moran, Notes on Continuous Stochastic Phenomena. *Biometrika* **37**, 17–23 (1950).

3. H. Li, C. A. Calder, N. Cressie, Beyond Moran’s I: Testing for Spatial Dependence Based on the Spatial Autoregressive Model. *Geogr. Anal.* **39**, 357–375 (2007).

4. J. Durbin, G. S. Watson, Testing for Serial Correlation in Least Squares Regression: I. *Biometrika* **37**, 409–428 (1950).
